# A novel susceptibility locus in the *IL12B* region is associated with the pathophysiology of Takayasu arteritis through IL-12p40 and IL-12p70 production

**DOI:** 10.1186/s13075-017-1408-8

**Published:** 2017-09-06

**Authors:** Toshiki Nakajima, Hajime Yoshifuji, Masakazu Shimizu, Koji Kitagori, Kosaku Murakami, Ran Nakashima, Yoshitaka Imura, Masao Tanaka, Koichiro Ohmura, Fumihiko Matsuda, Chikashi Terao, Tsuneyo Mimori

**Affiliations:** 1 Department of Rheumatology and Clinical Immunology, Graduate School of Medicine, Kyoto University, 54 Shogoin-Kawahara-cho, Sakyo-ku, Kyoto, 606-8507 Japan; 2 Center for Genomic Medicine, Graduate School of Medicine, Kyoto University, Kyoto, Japan; 3 Department of the Advanced Medicine for Rheumatic Diseases, Graduate School of Medicine, Kyoto University, Kyoto, Japan

**Keywords:** Takayasu arteritis, Vasculitis, Interleukin-12, Single nucleotide polymorphism, Monocytes

## Abstract

**Background:**

A previous study revealed the association between susceptibility to Takayasu arteritis (TAK) and a single nucleotide polymorphism (SNP) rs6871626 located in *IL12B*, which encodes interleukin (IL)-12p40, a common component of IL-12p70 and IL-23. We investigated the expression of these cytokines in patients with TAK, stratifying them into those with or without the risk allele at the rs6871626 SNP.

**Methods:**

Plasma levels of IL-12p40, IL-12p70, and IL-23 were quantified in 44 patients with TAK and 19 healthy controls (HCs) by enzyme-linked immunosorbent assays. Monocytes were obtained from 20 patients with TAK and 14 HCs, treated with interferon-γ (IFN-γ) and lipopolysaccharide, and then supernatant cytokines were quantified. In addition, the ratio of IFN-γ^+^ or IL-17A^+^ cells to CD4^+^ T cells was measured by flow cytometric analysis of peripheral blood mononuclear cells.

**Results:**

The levels of plasma IL-12p40, plasma IL-12p70, and supernatant IL-12p70 were significantly higher in patients with TAK than in HCs, whereas there were no significant differences in the levels of plasma IL-23, supernatant IL-23, or supernatant IL-12p40. The levels of plasma IL-12p70, supernatant IL-12p40, and supernatant IL-12p70 were significantly higher in patients with the risk allele than in those without. The ratio of CD4^+^IFN-γ^+^ cells was significantly higher in patients with the risk allele, whereas CD4^+^IL-17A^+^ cells showed no differences.

**Conclusions:**

The rs6871626 SNP in *IL12B* may influence the increased expression of IL-12p40 and IL-12p70. These enhanced cytokines might play roles in the pathophysiology of TAK.

**Electronic supplementary material:**

The online version of this article (doi:10.1186/s13075-017-1408-8) contains supplementary material, which is available to authorized users.

## Background

Takayasu arteritis (TAK), which was first reported in 1908 [1], is a type of large vessel vasculitis characterized by granulomatous arteritis of all artery layers and affects the aorta, its main branches, and pulmonary arteries. Stenosis, occlusion, and aneurysm of the affected arteries can occur during the progression of TAK and lead to organ failure including aortic regurgitation (AR), stroke, intermittent claudication, and renal failure. About 80–90% of patients with TAK are female and the age of onset of TAK is usually between 10 and 40 years [2]. Although glucocorticoids are mainly used to treat TAK, more effective and less toxic therapies are desired because relapses often occur after tapering the dose of glucocorticoids [3], and their side effects can be serious. Understanding of the pathophysiology of TAK is needed to improve its treatment, although this has not been fully revealed.

Some studies have reported that innate immunity, cytokines, and genomic factors are involved in the pathophysiology of TAK. First, innate immunity plays a role in tissue damage in patients with TAK. Seko et al. reported overexpression of costimulatory molecules (Fas and 4-1-BB ligand) and major histocompatibility class I chain-related A in the aortic tissue of patients with TAK [4]. Second, it has been reported that the blood concentrations of cytokines, such as interleukin (IL)-6 [5, 6], IL-8 [5], IL-12 [7], IL-18 [5, 6], IL-23 [8], and tumor necrosis factor-α [6], are elevated in patients with TAK, and some of them correlate with the disease activity of TAK [5, 7, 8]. Moreover, IL-6, IL-12, IL-17, and interferon-γ (IFN-γ) are highly expressed in the aortic tissues in patients with TAK [9]. These reports indicate that these cytokines play roles in the pathophysiology of TAK. Third, susceptibility to TAK is associated with certain genetic factors. For example, human leukocyte antigen (HLA)-B*52 is associated with the onset of TAK [10].

We previously reported that a single nucleotide polymorphism (SNP), rs6871626, in the *IL12B* region is associated with TAK, and that the risk allele at this SNP is correlated with clinical symptoms such as AR [11]. Furthermore, Matsumura et al. reported that the disease activity of TAK is more severe in patients with risk alleles at the rs6871626 SNP than in patients without risk alleles [12], but the mechanism underlying how the risk allele exacerbates the disease activity of TAK has not been revealed. *IL12B* encodes IL-12p40, a common subunit of IL-12p70 and IL-23. There have been some reports of IL-12p70 [5, 7] and IL-23 [5, 8] in TAK, but some of the results are inconsistent. Moreover, there have been no reports about the concentration of IL-12p40 in patients with TAK. To elucidate how these cytokines play roles in the pathophysiology of TAK, we examined the expression of IL-12p40, IL-12p70, and IL-23 in patients with TAK and the influence of the rs6871626 SNP on their production.

## Methods

### Patients

We recruited 44 patients with TAK who were being treated at Kyoto University Hospital and 19 healthy controls (HCs) (Table 1). TAK was classified according to the 1990 American College of Rheumatology criteria [2]. All participants provided written informed consent. Plasma was collected from all patients with TAK and from HCs, and stored at − 80 °C. This study was approved by the Ethics Committee of Kyoto University Graduate School and Faculty of Medicine (G412).

**Table 1:** Profiles of patients with TAK and healthy controls

	Risk group (*n* = 31)	Non-risk group (*n* = 13)	Healthy controls (*n* = 19)	*P* value
Age (years)	48.1 ± 14.6	40.3 ± 12.1	35.4 ± 9.3	<0.01^a^
Sex (male/female)	1/30	0/11	1/18	1.00^b^
Duration of disease (years)	16.8 ± 11.4	15.8 ± 11.1	―	0.97^c^
Average dose of glucocorticoids (mg/day)	6.3 ± 3.5	6.3 ± 4.0	―	0.64^c^
Average CRP (mg/dL)	0.43 ± 0.77	0.14 ± 0.21	―	0.052^c^
Average ESR (mm/h)	22.6 ± 17.7	20.4 ± 20.9	―	0.69^c^
Use of immunosuppressants	8/31 (25.8%)	4/13 (30.8%)	―	0.73^b^
Use of biologics	2/31 (6.5%)	0/13 (0%)	―	1.00^b^

Values are average ± standard deviation or number of patients. Immunosuppressants: methotrexate (*n* = 6; *n* = 4 in the risk group and 2 in the non-risk group) and azathioprine (*n* = 6; *n* = 4 in the risk group and 2 in the non-risk group). Biologics: tocilizumab (*n* = 2)

*TAK* Takayasu arteritis, *CRP* C-reactive protein, *ESR* erythrocyte sedimentation rate

^a^Statistical analysis performed by one-way analysis of variance.

^b^Statistical analysis performed by Fisher’s exact test

^c^Statistical analysis performed by Welch’s *t* test

### Stimulation of monocytes/macrophages

We employed the protocol of Johnston et al. [13]. Briefly, whole blood was collected from Patients with TAK (*n* = 20) and HCs (*n* = 14), and then monocytes were separated with a RosseteSep® human Monocyte Cell (StemCells, Vancouver, BC, Canada). Monocytes were incubated for 42 h at 37 °C in RPMI-1640 (Gibco, Waltham, MA, USA) containing 10% fetal calf serum (FCS), 5 ng/mL IL-10 (R&D Systems, Minneapolis, MN, USA), 5 ng/mL IL-6 (R&D Systems), and 10 ng/mL macrophage colony stimulating factor (PeproTech, Rocky Hill, NL, USA). Interferon-γ (IFN-γ; 50 ng/mL; R&D Systems) was added, and the monocytes were incubated for another 30 h. Then, the monocytes were incubated in RPMI-1640 containing 10% FCS with or without 1 μg/mL lipopolysaccharide (LPS; Sigma-Aldrich, St. Louis, MO, USA) for 12 h at 37 °C. The culture supernatant was then collected and stored at − 80 °C. In this protocol, we added IL-10, which is generally considered to promote monocytes to differentiate into M2 macrophages. However, we are satisfied that the monocytes were differentiated into M1 macrophages because we later added LPS, which strongly promotes M1 differentiation. We strictly followed the protocol that Johnston et al. used in their study on psoriasis, in which they showed the association of the *IL12B* SNP with the IL-12/23 production from macrophages [13].

### Cytokine measurements

IL-12p40, IL-12p70, and IL-23 in culture supernatants and plasma were measured with Human IL-12/IL-23 p40, Human IL-12, and Human IL-23 DuoSet enzyme-linked immunosorbent assays (R&D Systems), respectively, according to the manufacturer’s protocols.

### Flow cytometry

Whole blood was obtained from patients with TAK (*n* = 20) and HCs (n = 14), and peripheral blood mononuclear cells (PBMCs) were separated with Ficoll-Paque PLUS (GE healthcare, Pittsburgh, PA, USA). Some PBMCs were stained with anti-CD3, anti-CD4, anti-CXCR3, and anti-CCR6 antibodies or isotype controls and analyzed with a FACSCalibur platform (Becton, Dickinson and Company; BD, Franklin Lakes, NJ, USA).

To analyze intracellular cytokines, 4 × 10^6^ PBMCs were incubated in RPMI-1640 containing 10% FCS, 20 ng/mL phorbol myristate acetate (Sigma), 0.70 μg/mL ionomycin (Funakoshi, Tokyo, Japan) and Golgi-Stop (BD) for 5 h at 37 °C and then stained with anti-CD3, anti-CD4, anti-CCR6 antibodies or isotype controls. After fixation with BD Cytofix/Cytoperm (BD) overnight, PBMCs were stained with anti-IFN-γ or anti-IL-17A antibodies, or isotype controls and then analyzed using FACSCalibur. Flow cytometric data analysis was performed with FlowJo Ver. 6.0 (FlowJo LLC, Ashland, OR, USA). All antibodies and isotype controls were purchased from BD.

### Statistical analysis

Statistical analysis was performed with R 3.3.1 statistical software (R Foundation for Statistical Computing, Vienna, Austria). One-way analysis of variance (ANOVA), Welch’s *t* test, and Fisher’s exact test were used to analyze clinical variables. The Brunner-Munzel test and Spearman’s rank correlation coefficient were used to analyze experimental data. A *p* value less than 0.05 was considered as statistically significant and a *p* value between 0.05 and 0.15 was considered as a tendency.

## Results

### Plasma concentration of cytokines

**Fig. 1:**
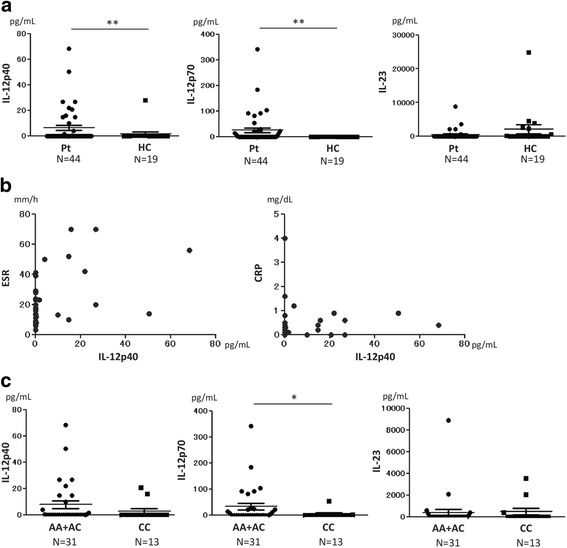
Plasma concentrations of IL-12p40, IL-12p70, and IL-23. **a** Plasma concentrations of IL-12p40 and IL-12p70 were significantly higher in patients (Pt) with Takayasu arteritis (TAK) than in healthy controls (HC) (*p* < 0.01 and *p* < 0.01, respectively). **b** Plasma concentrations of IL-12p40 in patients with TAK were positively correlated with erythrocyte sedimentation rate (ESR) (rho = 0.46, *p* < 0.01) and C-reactive protein (CRP) (rho = 0.31, *p* = 0.042). **c** Plasma concentrations of IL-12p70 were significantly higher in the risk group (AA + AC) than in the non-risk group (CC) (*p* = 0.012); A adenine, C cytosine. Statistical analysis was performed using a Brunner-Munzel test (**a** and **c**) and Spearman’s rank correlation coefficient (**b**). *p < 0.05 and ** *p* < 0.01

The plasma concentrations of both IL-12p40 and IL-12p70 were significantly higher in Patients with TAK than in HCs (*p* < 0.01 and *p* < 0.01, respectively). There were no significant differences in the plasma concentrations of IL-23 between patients with TAK and HCs (Fig. 1a).

We tested the correlation between cytokine levels and clinical parameters, and identified positive correlation between IL-12p40 and the erythrocyte sedimentation rate (ESR; rho = 0.46, *p* < 0.01), and between IL-12p40 and C-reactive protein (CRP; rho = 0.31, *p* = 0.042) (Fig. 1b). Age did not influence the production of IL-12p40, IL-12p70, or IL-23 (data not shown). There was weak positive correlation between IL-12p40 and IL-12p70 (rho = 0.33, *p* = 0.027), whereas there was no correlation between IL-12p40 and IL-23 (Additional file 1: Supplement 1).

Because adenine (A) at the rs6871626 SNP increases the risk of TAK compared with cytosine (C), patients with TAK were stratified into two groups, a risk group including patients with AA or AC at rs6871626 (*n* = 31) and a non-risk group including patients with CC at rs6871626 (*n* = 13). We found that the concentration of IL-12p70 was significantly higher in the risk group than in the non-risk group (*p* = 0.012). The concentration of IL-12p40 had a tendency to be higher in the risk group than in the non-risk group, although the difference was not statistically significant (*p* = 0.13). There were no differences in the concentrations of IL-23 between the risk and non-risk groups (Fig. 1c).

### Analysis of cytokine production by monocytes/macrophages

**Fig. 2:**
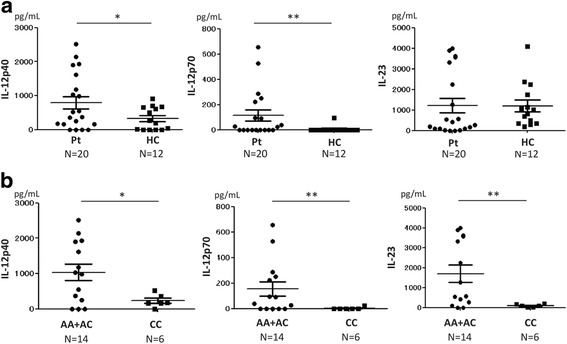
Cytokine concentrations in culture supernatants of monocytes/macrophages stimulated with interferon- γ and lipopolysaccharide. **a** Concentrations of IL-12p40 and IL-12p70 in patients with Takayasu arteritis (Pt) were significantly higher than in healthy controls (HC) (*p* = 0.049 and *p* < 0.01, respectively). **b** Concentrations of IL-12p40, IL-12p70, and IL-23 were significantly higher in the risk group (AA + AC) than in the non-risk group (CC) (*p* = 0.025, p < 0.01, and *p* < 0.01, respectively); A adenine, C cytosine. Statistical analysis was performed using the Brunner-Munzel test. *p < 0.05 and **p < 0.01

Because IL-12p40 is mainly produced by monocytes and macrophages, we collected monocytes from patients with TAK, induced their differentiation into macrophages, stimulated them with LPS, and measured the concentrations of IL-12p40, IL-12p70, and IL-23 in the culture supernatant. IL-12p40 and IL-12p70 levels in the culture supernatants of patient monocytes/macrophages were significantly higher than those of HC monocytes/macrophages (*p* = 0.049 and *p* < 0.01, respectively) (Fig. 2a). There were no significant differences in the levels of IL-23 between patients with TAK and HCs.

After stratifying patients with TAK into the risk group (*n* = 14) and the non-risk group (*n* = 6), the levels of IL-12p40, IL-12p70, and IL-23 were higher in the culture supernatants of the risk group than in those of the non-risk group (*p* = 0.025, *p* < 0.01 and *p* < 0.01, respectively) (Fig. 2b). Control monocytes/macrophages incubated without LPS did not produce any of these cytokines (data not shown).

### Analysis of helper T cell subsets

**Fig. 3:**
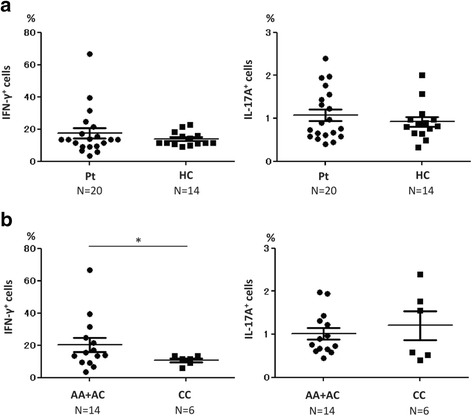
Proportions of T helper (Th)1 and Th17 cells. **a** There were no significant differences between patients with Takayasu arteritis (Pt) and healthy controls (HC) in the proportions of interferon-γ (IFN-γ)^+^ cells (Th1) and IL-17A^+^ cells (Th2). **b** The proportion of IFN-γ^+^ cells (Th1) was significantly larger in the risk group (AA + AC) than in the non-risk group (CC) (*p* = 0.037); A adenine, C cytosine. Statistical analysis was performed using the Brunner-Munzel test. *p < 0.05

Because IL-12p70 is essential for differentiation of T helper (Th)1 cells, and IL-23 is needed for maintenance of Th17 cells, we examined the proportion of Th1 and Th17 cells in the peripheral blood of patients with TAK and HCs. Surprisingly, there were no differences between patients with TAK and HCs in the proportions of IFN-γ^+^ cells (Th1) and IL-17A^+^ cells (Th17) CD3^+^CD4^+^ cells (Fig. 3a). When we stratified patients with TAK into the risk group (*n* = 14) and the non-risk group (*n* = 6), the proportion of IFN-γ^+^ cells was significantly larger in the risk group (*p* = 0.037), whereas there were no differences in the proportion of IL-17A^+^ cells between the risk and non-risk groups (Fig. 3b).

### Analysis of IFN-γ-producing CCR6^+^ cells

**Fig. 4:**
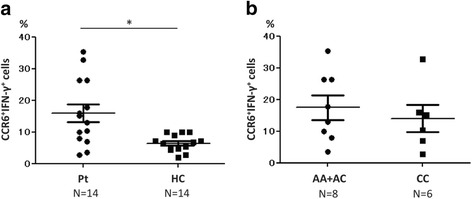
Proportion of CCR6^+^ interferon-γ (IFN-γ)^+^ cells among CD3^+^CD4^+^ cells. **a** The proportion of CCR6^+^IFN-γ^+^ cells was larger in patients with Takayasu arteritis (Pt) than in healthy controls (HC) (*p* = 0.012). **b** There were no significant differences between the risk group (AA + AC) and the non-risk group (CC); A adenine, C cytosine. Statistical analysis was performed using the Brunner-Munzel test. *p < 0.05

Recently, IFN-γ producing cells were found among CCR6^+^ cells and were named “non-classic” Th1 cells [14]. We analyzed IFN-γ^+^ cells among CD3^+^CD4^+^CCR6^+^ cells and found that the proportion of these cells was significantly larger in patients with TAK than in HCs (Fig. 4a). Additionally, the proportion of IFN-γ-producing Th17 cells had a tendency to be larger in the risk group than in the non-risk group, although the difference was not statistically significant (Fig. 4b).

## Discussion

In the present study, the levels of IL-12p70 in plasma and the culture supernatants of stimulated monocytes/macrophages were significantly higher in patients with TAK than in HCs, and there were no significant differences in IL-23 levels in plasma or in the culture supernatants of stimulated monocytes/macrophages between patients with TAK and HCs (Figs. 1a and 2a). These results are consistent with those reported by Verma et al. [7]. In contrast, Park et al. [6] and Tamura et al. [15] reported that IL-12p70 was not elevated in the serum of patients with TAK. This discrepancy may be because, as in our study, Verma et al. measured plasma IL-12p70, whereas Park et al. and Tamura et al. measured serum IL-12p70. In the studies by Park et al. and Tamura et al., the average levels of IL-12p70 were low in comparison with those found in our study and that of Verma et al. (10.2 and 2.1 vs 90.6 and 24.5 pg/mL). We suspect that these differences are associated with differences in the cytokine measurement methods (for example, the samples types, storage temperatures and assay kits used). Misra et al. reported that the serum IL-23 level was higher in patients with TAK than in HCs [8], which also conflicts with our data and those of Verma et al. In Misra’s report, the level of IL-23 appeared to decrease after treatment of patients with TAK. However, the disease activity in the patients in our cross-sectional study was relatively low. This may be a reason why we detected no differences in the IL-23 concentration between patients with TAK and HCs in the present study.

There have been no reports on the concentration of IL-12p40 in patients with TAK, although Tripathy et al. reported that IL-12p40 mRNA was more highly expressed in patients with TAK than in HCs [16]. To the best of our knowledge, this is the first report on the IL-12p40 protein level in patients with TAK. In our study, the level of IL-12p40 was significantly more elevated in plasma from patients with TAK than in HCs (Fig. 1a) and monocytes/macrophages derived from patients with TAK produced significantly more IL-12p40 than those derived from HCs (Fig. 2a). These results may suggest that IL-12p40 is involved in the pathophysiology of TAK. IL-12p40 has been reported to be highly expressed in the peripheral blood of patients with inflammatory bowel disease (IBD) [17] and in the intestinal mucosa of patients with ulcerative colitis (UC) [18]. We have previously reported a high rate of co-occurrence between TAK and UC, and that the two diseases share several genetic backgrounds [19]. These findings suggest that TAK and IBD may have a common pathophysiology. Thus, drugs effective for IBD may also be effective for TAK. In fact, azathioprine [20] and infliximab [21] are reported to be effective for the treatment of TAK. Because ustekinumab, a neutralizing antibody against IL-12p40, was reported to be effective for Crohn’s disease [22], ustekinumab is expected to be effective for TAK. We previously reported a pilot study of the treatment of TAK with ustekinumab, which had a favorable result [23]. Because IL-12p40 was correlated with ESR and CRP in the present study (Fig. 1b), IL-12p40 could be a disease activity marker of TAK, in a similar way to IBD [17].

When we analyzed the cytokine production in patients with TAK by stratifying them into those with or without the risk allele at the rs6871626 SNP, which is correlated with susceptibility of TAK [11], the plasma concentration of IL-12p70 was significantly higher in the risk group than in the non-risk group (Fig. 1c). The plasma levels of IL-12p40 in patients with TAK were positively correlated with ESR and serum CRP levels (Fig. 1b). This suggested that the disease activity was correlated with the levels of IL-12p40 in the patients with TAK. In our study, the disease activity of the patients was relatively low due to the treatments that the patients had already received. That might be the reason why we could not detect significant differences in the plasma concentrations of IL-12p40 between the risk group and the non-risk group, although the IL-12p40 levels in the risk group tended to be higher than those in the non-risk group.

Monocytes/macrophages derived from the risk group produced more IL-12p40, IL-12p70, and IL-23 than those derived from the non-risk group (Fig. 2b). This finding suggests that the rs6871626 SNP influences cytokine production by monocytes/macrophages. Because rs6871626 is located in the non-coding region of *IL12B*, it may regulate IL-12p40 expression. According to the Ensembl database (http://asia.ensembl.org/index.html), rs6871626 is transcribed into non-coding RNA (ncRNA). Because some ncRNAs regulate gene expression [24], rs6871626 may have a similar effect. A study has reported that rs6871626 is also a risk SNP for IBD [25]. The evidence that rs6871626 is associated with the incidence of both TAK and IBD implies that rs6871626 contributes to the regulation of IL-12p40 gene expression.

Because IL-12p70 is essential for Th0 cells to differentiate into Th1 cells, the proportion of Th1 cells was expected to be larger in patients with TAK than in HCs if the level of IL-12p70 was elevated in patients with TAK. Unexpectedly, in our study, we could not detect significant differences in the proportions of IFN-γ^+^ cells (Th1) and IL-17A^+^ cells (Th17) to CD3^+^CD4^+^ cells between patients with TAK and HCs (Fig. 3a). In our surface marker examinations, there was a negative correlation between the proportion of CXCR3^+^ cells, which are also regarded as Th1 cells, and the dose of glucocorticoids (Additional file 1: Supplement 2), which suggests that treatment of TAK might influence the activity of Th1 cells and, consequently, decrease the proportion of Th1 cells in patients with TAK.

The proportion of IFN-γ^+^ cells (Th1) was significantly larger in the risk group than in the non-risk group (Fig. 3b). This suggests that the differentiation from Th0 cells to Th1 cells may be promoted more strongly in patients with TAK who have the risk allele than in patients with TAK without the risk allele. Conversely, there were no differences in the proportion of IL-17A^+^ cells (Th17) between patients with TAK and HCs (Fig. 3a). In a report by Misra et al., the proportion of IL-17A^+^ cells was elevated in patients with TAK [8], which is inconsistent with our data. Again, this result may be because the TAK disease activity was relatively low in our study.

Annunziato et al. reported that some CCR6^+^ cells, called “non-classic” Th1 cells [12], do not secrete IL-17, but rather IFN-γ upon stimulation with IL-12p70 [26]. Harbour et al. reported that non-classic Th1 cells are essential to establish experimental colitis in mice [27]. Cosmi et al. reported that the number of non-classic Th1 cells increases in the synovial fluid of patients with juvenile idiopathic arthritis [28]. In our study, the proportion of IFN-γ-producing CCR6^+^ cells was significantly larger in patients with TAK than in HCs (Fig. 4a). This finding suggests that non-classic Th1 cells induced by IL-12p70 might play a role in the pathophysiology of TAK.

There are major limitations in our study. Because most of the patients with TAK in our study had been treated and the disease activity of TAK was low, the results may not reflect the cytokine profiles at the onset of TAK. Moreover, because TAK is a rare disease, the number of patients enrolled in our study was relatively small. The small number of participants might affect some of the results, especially after stratification into the risk and non-risk groups (type II statistical error). Recruitment of more patients and following their changes in cytokine concentrations during treatment should help to resolve these issues.

## Conclusions

The levels of IL-12p40 and IL-12p70 cytokines in plasma and culture supernatants of stimulated monocytes/macrophages were higher in patients with TAK than in HCs. When patients were stratified according to the presence/absence of the allele at the rs6871626 SNP, a risk factor for TAK, these cytokines were elevated in at-risk patients compared with non-risk patients. Together, these results suggest that the rs6871626 SNP located in the *IL12B* region might influence the production of IL-12p40 and IL-12p70, and that these cytokines might play important roles in the pathophysiology of TAK.

## Additional file

Additional file 1: Supplement 1. Correlations of the plasma concentration of IL-12p40 with those of IL-12p70 and IL-23. **a** The plasma concentration of IL-12p40 was correlated with that of IL-12p70 (rho = 0.33, *p* = 0.027). **b** There were no correlation between the plasma concentrations of IL-12p40 and IL-23. **Supplement 2.** Correlation between the proportion of CXCR3^+^ cells among CD3^+^CD4^+^ cells and the dose of glucocorticoids. The proportion of CXCR3^+^ cells among CD3^+^CD4^+^ cells was negatively correlated with the dose of glucocorticoids (rho = − 0.63 and *p* < 0.01). Statistical analysis was performed using Spearman’s rank correlation coefficient. (DOCX 34 kb)

## Abbreviations

AR: Aortic regurgitation
CCR: CC Chemokine receptor
CRP: C-reactive protein
CXCR: CXC chemokine receptor
ESR: Erythrocyte sedimentation rate
FCS: Fetal calf serum
HC: Healthy control
HLA: Human leukocyte antigen
IBD: Inflammatory bowel disease
IFN: Interferon
IL: Interleukin
LPS: Lipopolysaccharide
PBMC: Peripheral blood mononuclear cell
SNP: Single nucleotide polymorphism
TAK: Takayasu arteritis
Th: T helper
UC: Ulcerative colitis
