# Supplementary material for: A novel susceptibility locus in the IL12B region is associated with the pathophysiology of Takayasu arteritis through IL-12p40 and IL-12p70 production
Source: Arthritis Res Ther. 2017 Sep 6;19:197. doi: 10.1186/s13075-017-1408-8 (PMC5585951; doi:10.1186/s13075-017-1408-8)
Supplement: Additional file 1: Supplement 1. — Correlations of the plasma concentration of IL-12p40 with those of IL-12p70 and IL-23. a The plasma concentration of IL-12p40 was correlated with that of IL-12p70 (rho = 0.33, p = 0.027). b There were no correlation between the plasma concentrations of IL-12p40 and IL-23. Supplement 2. Correlation between the proportion of CXCR3+ cells among CD3+CD4+ cells and the dose of glucocorticoids. The proportion of CXCR3+ cells among CD3+CD4+ cells was negatively correlated with the dose of glucocorticoids (rho = − 0.63 and p < 0.01). Statistical analysis was performed using Spearman’s rank correlation coefficient. (DOCX 34 kb) [file 13075_2017_1408_MOESM1_ESM.docx]

**Additional file**

Supplement 1


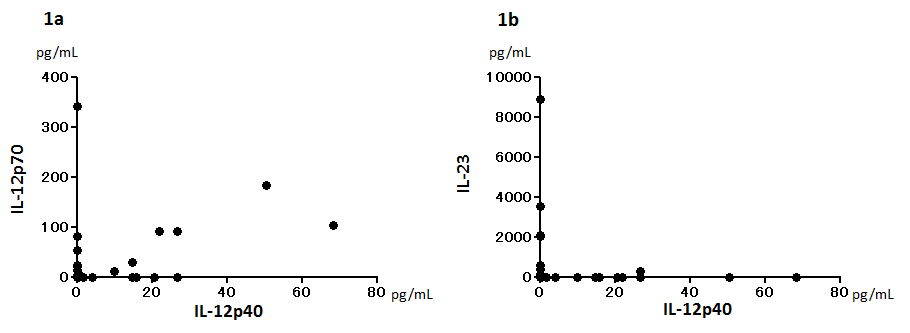


**Correlations of the plasma concentration of IL-12p40 with those of IL-12p70 and IL-23. a** The plasma concentration of IL-12p40 was correlated with that of IL-12p70 (rho = 0.33, p = 0.027). **b** There were no correlations between the plasma concentrations of IL-12p40 and IL-23.

Supplement 2


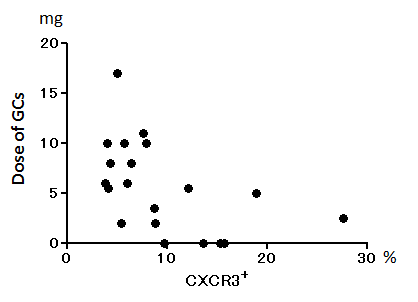


**Correlation between the proportion of CXCR3^+^ cells among CD3^+^CD4^+^ cells and the dose of glucocorticoids.** The proportion of CXCR3^+^ cells among CD3^+^CD4^+^ cells was negatively correlated with the dose of glucocorticoids (rho = −0.63 and p < 0.01). Statistical analysis was performed using a Spearman’s rank correlation coefficient.
